# Supplementary material for: SIPA1 Is a Modulator of HGF/MET Induced Tumour Metastasis via the Regulation of Tight Junction-Based Cell to Cell Barrier Function
Source: Cancers (Basel). 2021 Apr 6;13(7):1747. doi: 10.3390/cancers13071747 (PMC8038768; doi:10.3390/cancers13071747)
Supplement: Supplementary file 1 [file cancers-13-01747-s001.pdf]

cancers

MDPI

Table S1. Primers for conventional RT-PCR, QPCR and ribozyme synthesis.

PRIMER NAME	PRIMER SEQUENCE (5′-3′)
**GAPDHF8**	GGCTGCTTTTAACTCTGGTA
**GAPDHR8**	GACTGTGGTCATGAGTCCTT
**GAPDHF1**	AAGGTCATCCATGACAACTT
**GAPDHZR1**	ACTGAACCTGACCGTACAGCCATCCACAGTCTTCTG
**SIPA1F1**	AGAACTCGCTGTCACCAC
**SIPA1ZR**	ACTGAACCTGACCGTACATACTGATGGCTTGGCTGT
**CLDN1F**	GAAGTGTATGAAGTGCTTGG
**CLDN1ZR**	ACTGAACCTGACCGTACAGACCTGCAAGAAGAAATA
**CLDN2F1**	AAGTCTTTGACTCCTTGCTG
**CLDN2ZR**	ACTGAACCTGACCGTACAGCCACAAAGATTGCTATCAC
**CLDN3F1**	ACTGCCACAGGACCTTCA
**CLDN3ZR**	ACTGAACCTGACCGTACAATGGTGATCTTGGCCTTG
**CLDN4F1**	GCAGCAACATTGTCACCT
**CLDN4ZR**	ACTGAACCTGACCGTACAGTACACCTTGCACTGCATC
**CLDN 5F1**	TTCCTGGACCACAACATC
**CLDN5ZR1**	ACTGAACCTGACCGTACACACCGAGTCGTACACTTTGC
**CLDN5F2**	TTCCTGGACCACAACATC
**CLDN5ZR2**	ACTGAACCTGACCGTACACACCGAGTCGTACACTTTGC
**CLDN6F1**	ACGTGCCCTCTGTGTCAT
**CLDN6ZR**	ACTGAACCTGACCGTACACTCCACACAGGTGGTACACT
**CLDN7F1**	ATAACCCTTTGATCCCTACC
**CLDN7ZR**	ACTGAACCTGACCGTACAACAGGAACAGGAGAGCAGT
**CLDN8F1**	GCTTGGAGAAGCTCTCTACTT
**CLDN8ZR**	ACTGAACCTGACCGTACAAGCTACTGCTCTTTTCGTTG
**CLDN9F1**	GTGCCCTCTGTGTCATTG
**CLDN9ZR**	ACTGAACCTGACCGTACATCCACACACGTGGTACACT
**CLDN10F1**	GTCTCCAACTGCAAGGACT
**CLDN10ZR**	ACTGAACCTGACCGTACAGCAAATATGGAACCAAAGAA
**CLDN11F1**	CCGGTGTGGCTAAGTACA
**CLDN11ZR**	ACTGAACCTGACCGTACACACACAGGGAACCAGATG
**CLDN12F1**	CTCTGCCTGATTGGAATG
**CLDN12ZR**	ACTGAACCTGACCGTACAACCTGCACTATTGACCAGAC
**CLDN14F1**	CACCCTACAGGCCCTACC
**CLDN14ZR**	ACTGAACCTGACCGTACAGTCTTTGTAGGCAGCTGGT
**CLDN15F1**	CCTGGGCGTCTACAACTG
**CLDN15ZR**	ACTGAACCTGACCGTACAATAGACATCAGCCGGACA
**CLDN17F1**	CTGCTTATTGGCATCTGTG
**CLDN17ZR**	ACTGAACCTGACCGTACATGAAGTTCCCAGAAGGTATG
**CLDN18F1**	GGATCATGTTCATTGTCTCA
**CLDN18ZR**	ACTGAACCTGACCGTACACATCCAGAAGTTAGTCACCAG
**CLDN19F1**	GGAGCCCTCTTCATCCTG
**CLDN19ZR**	ACTGAACCTGACCGTACAGTGCTTGGGTTGAAGAACT
**CLDN20F1**	AGCAAACTTTCTGGATCTGA
**CLDN20ZR**	ACTGAACCTGACCGTACACAGAAAATCATGCCAGAGAT
**CLDN22F1**	CAGGCTCTTGTAACCTCAAC
**CLDN22ZR**	ACTGAACCTGACCGTACATTGGATGACACAGGTTTG
**CLDN23F1**	CCGCCATCAAGTACTACAG
**CLDN23ZR1**	ACTGAACCTGACCGTACACCACCGAGTTGGTGTAGG
**CLDN24F1**	GTGGACGCACATGACTGT
**CLDN24ZR**	ACTGAACCTGACCGTACACAGGAGGAAGGTGAGCTG
**JAM1F2**	AACAAGATCACAGCTTCCTA
**JAM1ZR**	ACTGAACCTGACCGTACAACAAGTGTATGTCCCAGTGT

**JAM2F1**	CTGATAGGGGCTGTAAATCT
**JAM2ZR**	ACTGAACCTGACCGTACATAATGATGCAAGACAGTTCC
**JAM3F1**	TTGATGAGATTGTGATCGAG
**JAM3ZR**	ACTGAACCTGACCGTACAATCTTGCCTACTGGTACAGC
**ZO1ZF2**	TGGGTATGACACACATGGTA
**ZO1ZR2**	ACTGAACCTGACCGTACAGGTGGTACTTGCTCGTAA
**ZO2F1**	CAAAAGAGGATTTGGAATTG
**ZO2ZR1**	ACTGAACCTGACCGTACAGAGCACATCAGAAATGACAA
**ZO3F1**	CTGACATGGAGGAGCTGA
**ZO3ZR1**	ACTGAACCTGACCGTACAGCTTAGCTTCCCTTCTGACT
**AF6F**	ATCATCCACCACTGGAAC
**AF6ZR**	ACTGAACCTGACCGTACATCATCACTCCATGGAACTC
**OCLNZF**	G AATTCAAACCGAATCATTG
**OCLNZR**	ACTGAACCTGACCGTACATGAAGAATTTCATCTTCTGG

Z sequence 'ACTGAACCTGACCGTACA' highlighted in blue. Z-sequence presents on the specific primers containing a 5′ hairpin structure labelled with a fluorophore (FAM).

Table S2. Primary and secondary antibodies used in this study.

**Antibodies**	**Molecular weight (kDa)**	**Supplier**	**Product code**
Mouse anti SIPA1	112	Abcam	ab219085
Mouse anti GAPDH	37	Insight Biotechnology	sc-32233
Mouse anti AF6	200	Insight Biotechnology	sc-74433
Rabbit anti ZO1	220	Thermo Fisher Scientific	61-7300
Goat anti ZO2	160	Insight Biotechnology	sc-12386
Goat anti ZO3	80-160	Insight Biotechnology	sc-11475
Goat anti Claudin1	22	Insight Biotechnology	sc-17658
Rabbit anti Claudin5	23	Insight Biotechnology	sc-28670
Goat anti Claudin7	24	Insight Biotechnology	sc-17670
Goat anti Claudin9	23	Insight Biotechnology	sc-17672
Rabbit anti Claudin10	23/19	Insight Biotechnology	sc-25710
Rabbit anti Claudin11	20	Insight Biotechnology	sc-25711
Goat anti Claudin15	23	Insight Biotechnology	sc-17683
Rabbit anti Claudin19	22	Insight Biotechnology	sc-134618
Rabbit anti Claudin20	23.5	Novusbio	H00049861-D01P
Goat anti Claudin23	32	Insight Biotechnology	sc-87047
Mouse anti JAM1	36	Insight Biotechnology	sc-53623
Goat anti JAM2	54	Insight Biotechnology	sc-23002
Goat anti JAM3	38	Insight Biotechnology	sc-23006
Goat anti Occludin	60-82	Insight Biotechnology	sc-8145
Rabbit anti-mouse (whole molecule) IgG peroxidise conjugate	Dependent on primary	Sigma-Aldrich	A5278
Goat anti-rabbit (whole molecule) IgG peroxidise conjugate	Dependent on primary	Sigma-Aldrich	A0545
Rabbit anti-goat (whole molecule) IgG peroxidise conjugate	Dependent on primary	Sigma-Aldrich	A5420

Table S3. Top signalling pathways which were downregulated by SIPA1 knockdown on A549 cells. (Generated from: www.reactome.org.).

Pathway name	Entities				Reactions	
	found	ratio	p-value	FDR*	found	ratio
Signaling by SCF-KIT	6 / 51	0.004	4.86e-08	2.76e-05	21 / 36	0.003
CD28 co-stimulation	5 / 39	0.003	4.48e-07	1.27e-04	10 / 19	0.002
Interleukin-3, Interleukin-5 and GM-CSF signaling	5 / 50	0.003	1.50e-06	2.84e-04	10 / 38	0.003
Regulation of signaling by CBL	4 / 24	0.002	2.44e-06	3.44e-04	4 / 7	5.61e-04
ESR-mediated signaling	8 / 256	0.018	5.67e-06	6.26e-04	37 / 110	0.009
Signaling by Receptor Tyrosine Kinases	11 / 554	0.038	6.66e-06	6.26e-04	92 / 657	0.053
PECAM1 interactions	3 / 14	9.70e-04	2.31e-05	0.002	1 / 7	5.61e-04
Signaling by Interleukins	11 / 639	0.044	2.51e-05	0.002	52 / 490	0.039
Fc epsilon receptor (FCERI) signaling	7 / 235	0.016	3.10e-05	0.002	27 / 63	0.005
Costimulation by the CD28 family	5 / 97	0.007	3.61e-05	0.002	12 / 34	0.003
Generic Transcription Pathway	17 / 1,553	0.108	4.17e-05	0.002	61 / 824	0.066
EPHB-mediated forward signaling	4 / 51	0.004	4.60e-05	0.002	9 / 26	0.002
Regulation of KIT signaling	3 / 18	0.001	4.85e-05	0.002	3 / 8	6.41e-04
Signal Transduction	26 / 3,305	0.229	6.20e-05	0.002	240 / 2,303	0.184
Oxidative Stress Induced Senescence	5 / 114	0.008	7.70e-05	0.003	17 / 40	0.003
Fcgamma receptor (FCGR) dependent phagocytosis	6 / 193	0.013	9.45e-05	0.003	7 / 42	0.003
Signaling by Nuclear Receptors	8 / 385	0.027	1.02e-04	0.003	37 / 191	0.015
Cellular Senescence	6 / 199	0.014	1.12e-04	0.003	26 / 90	0.007
RNA Polymerase II Transcription	17 / 1,692	0.117	1.23e-04	0.004	61 / 885	0.071
CTLA4 inhibitory signaling	3 / 25	0.002	1.27e-04	0.004	2 / 5	4.00e-04
Developmental Biology	14 / 1,207	0.084	1.31e-04	0.004	109 / 511	0.041
PIP3 activates AKT signaling	7 / 316	0.022	1.95e-04	0.005	9 / 86	0.007
Cellular responses to stress	10 / 690	0.048	2.52e-04	0.006	45 / 227	0.018
EPHA-mediated growth cone collapse	3 / 33	0.002	2.87e-04	0.006	3 / 4	3.20e-04
Estrogen-dependent gene expression	5 / 154	0.011	3.09e-04	0.006	27 / 66	0.005

Table S4. Top signalling pathways which were up regulated by SIPA1 knockdown on A549 cells. (Generated from: www.reactome.org.).

Pathway name	Entities				Reactions	
	found	ratio	p-value	FDR*	found	ratio
MyD88:MAL(TIRAP) cascade initiated on plasma membrane	13 / 111	0.008	7.25e-11	5.41e-09	45 / 64	0.005
Toll Like Receptor TLR6:TLR2 Cascade	13 / 111	0.008	7.25e-11	5.41e-09	45 / 66	0.005
MAP kinase activation	11 / 69	0.005	9.09e-11	5.41e-09	24 / 32	0.003
Toll Like Receptor TLR1:TLR2 Cascade	13 / 114	0.008	1.00e-10	5.41e-09	45 / 66	0.005
Toll Like Receptor 2 (TLR2) Cascade	13 / 114	0.008	1.00e-10	5.41e-09	45 / 68	0.005
MyD88 cascade initiated on plasma membrane	12 / 94	0.007	1.53e-10	6.56e-09	30 / 58	0.005
Toll Like Receptor 5 (TLR5) Cascade	12 / 94	0.007	1.53e-10	6.56e-09	30 / 59	0.005
Toll Like Receptor 10 (TLR10) Cascade	12 / 94	0.007	1.53e-10	6.56e-09	30 / 59	0.005
Interleukin-17 signaling	11 / 77	0.005	2.85e-10	1.17e-08	24 / 35	0.003
Signaling by Interleukins	26 / 639	0.044	3.60e-10	1.30e-08	125 / 490	0.039
TRAF6 mediated induction of NFkB and MAP kinases upon TLR7/8 or 9 activation	12 / 102	0.007	3.81e-10	1.30e-08	30 / 48	0.004
Toll Like Receptor 3 (TLR3) Cascade	12 / 102	0.007	3.81e-10	1.30e-08	31 / 61	0.005
MyD88 dependent cascade initiated on endosome	12 / 103	0.007	4.25e-10	1.32e-08	30 / 63	0.005
Toll Like Receptor 7/8 (TLR7/8) Cascade	12 / 103	0.007	4.25e-10	1.32e-08	30 / 64	0.005
Signaling by Receptor Tyrosine Kinases	24 / 554	0.038	5.54e-10	1.61e-08	174 / 657	0.053
TRIF(TICAM1)-mediated TLR4 signaling	12 / 107	0.007	6.51e-10	1.76e-08	31 / 58	0.005
MyD88-independent TLR4 cascade	12 / 107	0.007	6.51e-10	1.76e-08	31 / 60	0.005
Toll Like Receptor 9 (TLR9) Cascade	12 / 107	0.007	6.51e-10	1.76e-08	30 / 67	0.005
Generic Transcription Pathway	41 / 1,553	0.108	7.51e-10	1.95e-08	279 / 824	0.066
Toll Like Receptor 4 (TLR4) Cascade	13 / 144	0.01	1.64e-09	4.10e-08	48 / 95	0.008
RNA Polymerase II Transcription	42 / 1,692	0.117	2.77e-09	6.64e-08	281 / 885	0.071
Gene expression (Transcription)	43 / 1,850	0.128	1.20e-08	2.88e-07	293 / 996	0.08
Nuclear Receptor transcription pathway	10 / 86	0.006	1.30e-08	2.99e-07	2 / 2	1.60e-04
Toll-like Receptor Cascades	13 / 184	0.013	2.89e-08	6.35e-07	53 / 182	0.015
VEGFA-VEGFR2 Pathway	11 / 126	0.009	4.33e-08	9.54e-07	37 / 77	0.006
